# Ion-Conducting Robust Cross-Linked Organic/Inorganic Polymer Composite as Effective Binder for Electrode of Electrochemical Capacitor

**DOI:** 10.3390/polym14235174

**Published:** 2022-11-28

**Authors:** Hui Gyeong Park, Jin Ju Jeong, Jae Hun Kim, Jung-Soo Lee

**Affiliations:** 1 Department of Chemical Engineering, Graduate School of Chosun University, 309 Pilmun-daero, Dong-gu, Gwangju 61452, Republic of Korea; 2 Department of Bio-Chemical Engineering, Chosun University, 309 Pilmun-daero, Dong-gu, Gwangju 61452, Republic of Korea

**Keywords:** poly(ionic liquids), polymeric binder, cross-linking, energy storage/conversion devices, pseudocapacitor

## Abstract

Poly(ionic liquid)s (PILs) are used in many electrochemical energy storage/conversion devices owing to their favorable physical properties. Therefore, PIL binders have been examined as polymeric binders for electrodes in energy storage systems (ESSs) and have shown superior performance. Several innovative technologies have been developed to improve the properties of polymers, with cross-linking being the most effective and easy strategy to achieve this. In this study, we designed a breakthrough complex cross-linking and composite technique that could successfully develop the physical properties of a polymer in a simple one-step process. Additionally, the technique could improve the thermal stability and mechanical properties of the polymer. The proposed polymeric binder showed better adhesion, higher capacitance, and good energy density with improved cyclic stability compared to that shown by conventional polyvinylidene fluoride (PVDF). This study revealed that cross-linked networks in polymeric binders are long-cycle-life features for electrochemical redox capacitors.

## 1. Introduction 

With advancements in technology, modern society demands the development of high-efficiency, high-density, and long-life energy storage devices, ranging from portable electronic devices to electric vehicles [1,2]. Fossil fuels have been used to produce energy for several decades [3]. Formulating and implementing strategies to develop clean and sustainable energy systems to reduce the consumption of fossil fuels and alleviate environmental pollution is of global significance [4,5].

Energy storage systems (ESS) play a prominent role in the proper utilization and storage of green energy [6,7,8,9,10,11,12]. Supercapacitors, which store energy by the adsorption of ions or surface redox reactions, are considered the most promising ESSs [13,14].

Supercapacitors offer advantages such as shorter charging/discharging times and higher power densities compared to those offered by lithium-ion batteries (LIBs) [15,16,17]. Electrochemical double-layer capacitors (EDLCs) store charges electrostatically at the interface between the electrode material and electrolyte [18,19]. In response to fast surface redox reactions, pseudocapacitors (PSEUDOs) store charges in the electrode, providing a capacitive-like electrochemical behavior [20,21,22,23]. The active materials of PSEUDO exhibit higher capacitances than those of EDLCs. However, such PSEUDOs often produce compromised rate capability and reversibility because they rely on faradaic redox reactions [24]. Most polymer binders only occupy a small proportion (<5 wt%) of commercial electrodes [25,26,27]. However, they play an important role in physically stabilizing electrodes. When used as binders, polymer materials should not chemically reject organic electrolytes and should instead maintain stable adhesive properties without undergoing side reactions, even in an electrochemical environment [28,29,30,31]. Although polyvinylidene fluoride (PVDF) is the most successful and widely used binder for conventional LIBs, it suffers from several limitations, such as having weak intermolecular interactions with active materials and showing an inability to support the fast ion transport required under high rates [32,33].

The homogeneous composite structure of pristine electrodes becomes disrupted due to substantial volume changes, leading to mechanical failure and capacity loss over repeated charge/discharge cycles. Several studies have pointed out that the cyclability-related problems of the most promising advanced electrode materials can be alleviated by using more efficient battery binders [34,35]. An ideal electrode matrix should (1) form strong interactions with active materials to maintain adhesion over cycles; (2) offer strong adhesion toward current collectors to prevent electrode delamination; (3) provide a continuous conductive network within the electrode; and (4) be electrochemically and chemically stable in harsh environments for batteries. A class of polymers that has recently garnered attention is polymeric ionic liquids (PILs), whose high electrochemical stability, conductivity, and good processability support their suitability for electrochemical applications [36,37,38,39,40,41,42]. They have been successfully incorporated in supercapacitors, electrochemical sensors, and a variety of energy devices. PILs can wrap powder components while still allowing ion flow, improving cyclability [43].

The cross-linking of polymer binders has been proposed as an effective and simple method for the volume expansion of electrodes [44,45,46]. Thermal and mechanical analyses that were conducted to examine some cross-linked polymers revealed increased glass transition and rheology in them, similar to those in permanently cross-linked polymer matrices [47]. 

In this study, we present nickel oxide (NiO) synthesized by the sol–gel method and a cross-linkable organic/inorganic hybrid polymer successfully obtained through the quaternization of poly(N-vinyl imidazole) (PVIm) with a unique functional group containing ethoxysilane [48]. We assumed that the durability would be improved through the formation of Si–O bonds between the polymer chains and the active material from introducing a cross-linkable silane group. We fabricated a pseudocapacitor with PVIm; the corresponding electrode could provide cycle stability through the simultaneous improvement in the ion conductivity and physical properties of the composite through cross-linking. Currently, the main trend is developing devices that can be stored while maintaining the cycle characteristics and also increasing the output energy; continuous research and development on PSEUDOs is expected to create new opportunities because they are located in an industry group. 

## 2. Experimental Section

### 2.1. Materials

Vinyl imidazole (Vm) (98%, Alfa Aesar, Haverhill, MA, USA), a,a′-azobisisobutyronitrile (AIBN) (98%, JUNSEI), (3-chloropropyl)triethoxysilane (3CPTES) (Sigma Aldrich， St. Louis， MI, USA), dimethylformamide (DMF) (99.5%, JUNSEI), acetone (extra pure grade, DUKSAN), methanol (extra pure grade, DUKSAN), nickel (II) nitrate hexahydrate (Ni(NO_3_)_2_·6H_2_O) (98%, Alfa Aesar), hexamethylenetetramine (HMTA) (99%, KANTO), carbon black (99.9%, Alfa Aesar), PVDF 1 wt% solution, and N-methyl-2-pyrrolidone (NMP) (99.5%, SAMCHUN, Seoul, Republic of Korea) were used without any further purification. AIBN was recrystallized from methanol.

### 2.2. Method

#### 2.2.1. Synthesis of Poly(Vinyl Imidazole) (PVIm)

**Figure 1:**
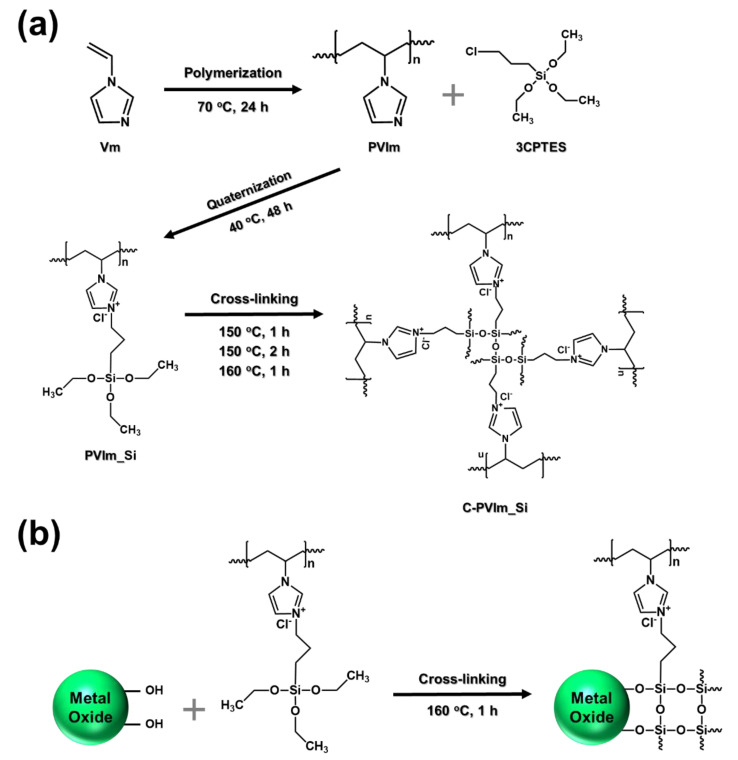
(**a**) Synthesis of C-PVIm_Si; (**b**) metal oxide surface reactions.

Vm was polymerized via free-radical polymerization, as shown in Figure 1. Initially, Vm and DMF were briefly added to a round-bottom flask equipped with a magnetic stirrer. AIBN was added to the flask, and the mixture was slowly heated to 70 °C under a nitrogen atmosphere. After radical polymerization for 24 h, the resulting yellow viscous solution was cooled to room temperature and precipitated with acetone; this was followed by filtration, which yielded a white powder. The vacuum was dried overnight at 40 °C. The white powder was dissolved in DMF, and the precipitation process was repeated twice. Finally, the resulting wet powder was dried overnight under vacuum at 60 °C to obtain a white powder of PVIm.

#### 2.2.2. Quaternization of PVIm with 3CPTES

PVIm_Si was synthesized via the nucleophilic substitution reaction (S_N_2) of PVIm and 3CPTES. This synthesis was conducted at molar ratios of 100:50, 100:70, and 100:100.

PVIm and 3CPTES (50, 10.7 mmol), (70, 14.9 mmol), (100, 21.2 mmol) and DMF (40 mL) were added to a 250 mL round-bottom flask equipped with a magnetic stirrer. The mixture was then stirred at 40 °C for 48 h. The resulting solution was precipitated with acetone, followed by filtration to a white powder. The white powder was dried overnight under vacuum at 40 °C, as shown in Figure 1. 

#### 2.2.3. Thermal Cross-Linked Poly(1-Vinyl-Propyltriethoxysilane Imidazolium)·Cl^−^ (C-PVIm_Si)∙Cl^−^

Finally, the prepared PVIm_Si films were placed in vacuum at 150, 150, and 160 °C for 1, 2, and 1 h, respectively, to conduct thermal cross-linking. We refer to the obtained sample as C-PVIm_Si.

#### 2.2.4. Synthesis of Ni(OH)_2_ Nanostructure

Using magnetic stirring, Ni(NO_3_)_2_·6H_2_O and HMTA were dissolved in deionized (DI) water. The mixed solution was then transferred into a 100 mL vial, maintained at 100 °C for 4 h, and then cooled naturally. The synthesized nickel hydroxide products were cleaned by washing with DI water. The prepared samples were then freeze-dried for 48 h [47]. 

#### 2.2.5. Synthesis of Porous NiO Nanostructure

Porous NiO nanostructure powders were obtained by annealing the as-prepared Ni(OH)_2_ samples at 300 °C for 2 h. NiO is generally known to exhibit excellent electrochemical properties, including pseudocapacitance [48,49].

### 2.3. Characterization

The chemical structures of the films were analyzed using Fourier-transform infrared spectroscopy (FTIR, Nicolet 6700, Thermo Scientific, Waltham, MA, USA) and proton nuclear magnetic resonance (^1^H-NMR spectra, JEOL-JNM-AL300, Tokyo, Japan) in dimethyl sulfoxide-d_6_ (DMSO-d_6_) at 300 MHz. The thermal properties of C-PVIm were investigated using differential scanning calorimetry (DSC, TA Instruments, DSC 25, New Castle, DE, USA) under a N_2_ atmosphere (in a temperature range of 25–180 °C and at a heating rate of 5 °C min^−1^) and thermogravimetric analysis (TGA, TA Instruments, SDT 650, New Castle, DE, USA) under a N_2_ atmosphere (in a temperature range of 25–1000 °C and at a heating rate of 10 °C min^−1^). To confirm the degree of chemical cross-linking, the gel content was determined using the solvent extraction method and was calculated using the following equation:

(1) $$\frac{M_{2}}{M_{1}}\times 100\%=\mathrm{gel}\;\mathrm{content},$$

where M_1_ is the weight of C-PVIm_Si, and M_2_ is the weight of the extracted solvent [50].

Based on the American Society for Testing and Materials (ASTM) standards, several 180° peel tests were performed using a universal testing machine (UTM, Shimadzu, AGS-10Knx, Tokyo, Japan). The samples were measured at room temperature (20 mn min^−1^).

The electrodes were morphologically and electrochemically characterized using scanning electron microscopy (SEM, Hitachi Regulus 8100, Tokyo, Japan).

### 2.4. Electrochemical Characterization

The working electrode was prepared by mixing an electroactive material (NiO, 85 wt%), carbon black (10 wt%), PVDF, and PVIm_Si (5 wt%) as a binder. The prepared slurry was measured in a nickel plate electrode, and the PVDF electrode binder was dried at 120 °C for 1 h to remove the NMP. The PVIm_Si binder was manufactured by drying at 120 °C for 1 h to remove the NMP, followed by drying for 1 h at 160 °C; this was the optimal cross-linking condition.

The electrochemical performance of NiO was evaluated using Pt foil as the counter electrode and a KOH solution (2 M) as the electrolyte. A voltage window of −0.05 to 0.4 V was observed at room temperature.

## 3. Results and Discussion

The experimental method is schematically illustrated in Figure 1. Some PVIm and 3CPTES were sequentially dispersed in NMP and heated to 40 °C for 48 h. To obtain the cross-linking rate, depending on the ratio of silane, polymerization was carried out by controlling the ratio of PVIm to silane (100:50, 100:70, and 100:100). The samples obtained from these processes were made into films and cross-linked at different temperatures and times (150, 150, and 160 °C for 1, 2, and 1 h, respectively). In addition, the structural formula after the cross-linking of NiO and PVIm_Si, shown in Figure 1b, confirmed the FT-IR peak shown in Figure S1.

**Figure 2:**
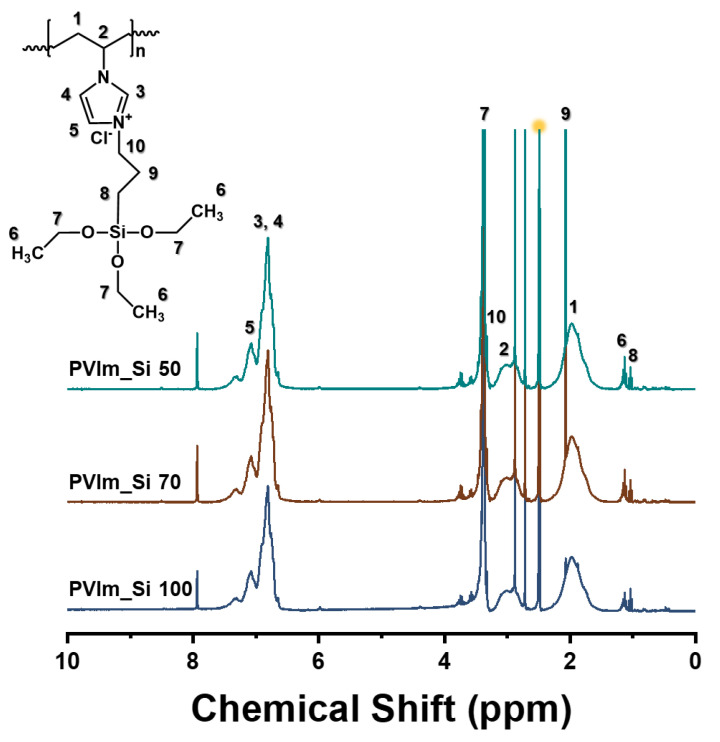
^1^H-NMR spectra of PVIm_Si.

Free radical polymerization was used to prepare PVIm, using AIBN as the initiator. PVIm_Si was prepared by the partial functionalization of PVIm with 3CPTES. This reaction proceeded smoothly owing to the weak steric hindrance and strong nucleophilicity of the 3-position nitrogen atom in the imidazole ring, as well as the high reactivity of 3CPTES. Figure 2 shows the ^1^H-NMR spectra of PVIm_Si 50, 70, and 100 in the range of 0–10 ppm, with DMSO-d_6_ (yellow dot) as the deuterated reagent. The peaks between 6.7 ppm (H_3,4_) and 7.6 ppm (H_5_) were ascribed to the proton of the imidazole ring. Meanwhile, the peaks at 1.6–2.3 ppm (H1) and 2.88–3.2 ppm (H_2_) corresponded to protons from the polymer backbone (CH_2_ and CH–). The peaks at 1.2 (H_6_) and 3.4 ppm (H_7_) were ascribed to the ethyl group (CH_3_, CH_2_) [51,52,53,54]. The ^1^H-NMR peaks of PVIm and 3CPTES used for quaternary substitution are shown in Figure S2.

**Figure 3:**
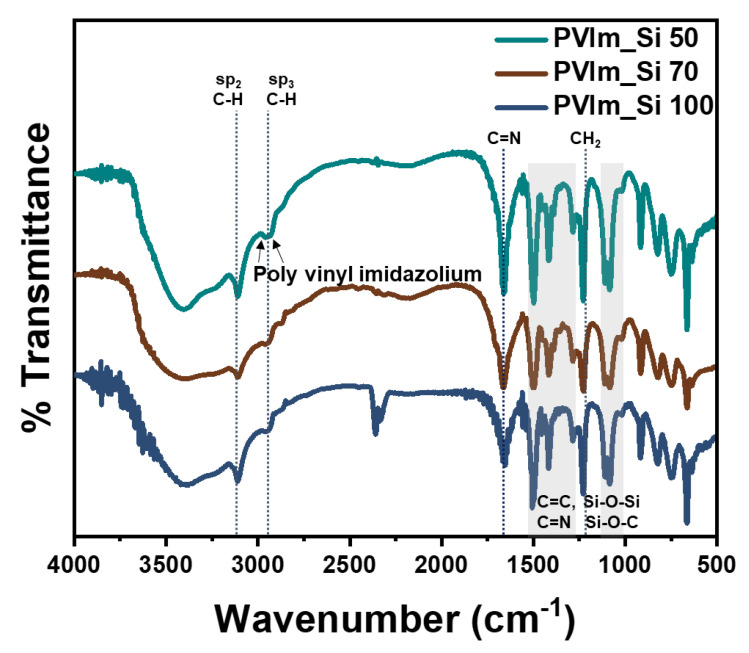
FT-IR spectra of PVIm_Si.

The chemical structure of the imidazolium-based binder was confirmed using FT-IR spectroscopy. Figure 3 shows the FT-IR of C-PVIm_Si; the broad absorption band at approximately 3018 and 2983 cm^−1^ can be attributed to the stretching vibrations of C-H (sp^2^) and C-H (sp^3^) groups, respectively [55]. The peaks at 2916 and 2846 cm^−1^ were attributed to the PVIm moieties [56,57]. The peak at 1750 cm^−1^ confirmed the presence of an aromatic C=N. The peaks at 1510 to 1276 cm^−1^ confirmed the presence of the imidazole ring [58]. The absorption bands at 1250 to 1084 cm^−1^ were attributed to Si-O-Si and Si-O-C stretching [59,60,61,62,63]. Finally, PVIm_Si was successfully synthesized [64]. Moreover, Figure S3 displays the FT-IR spectrum of C-PVIm_Si with peaks of interest marked.

**Figure 4:**
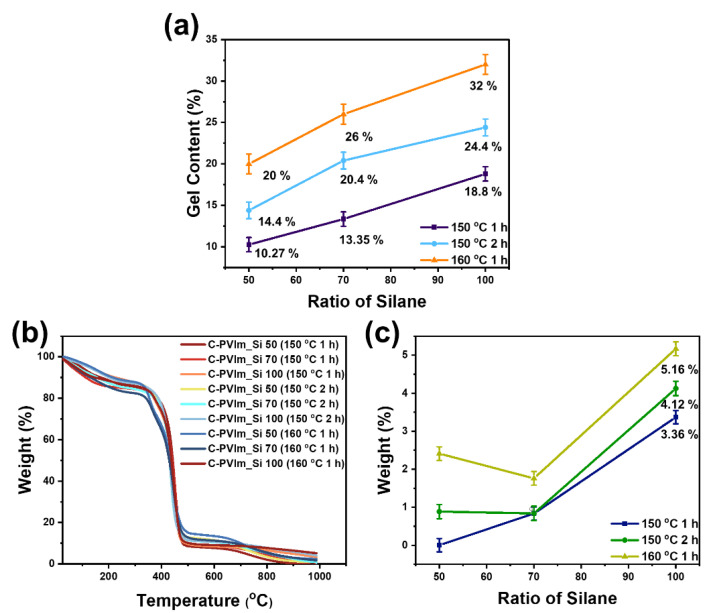
(**a**) Gel content of C-PVIm_Si; (**b**) TGA curves of C-PVIm_Si; (**c**) final weight based on TGA.

C-PVIm_Si was prepared via thermally induced cross-linking. It is well known that the thermal treatment of ethoxy silane groups leads to the formation of insoluble cross-linked networks (Si-O-Si) in the polymer matrix. The gel content as a function of the temperature is shown in Figure 4a. The results confirmed that the gel content significantly increased with the increasing ratio of quaternized silane in the PVIm matrix and thermal treatment temperature up to 160 °C. 

Excellent thermal stability is a fundamental requirement for PSEUDOs in practical applications. The thermal degradation behavior of the polymer binder was examined using TGA in a nitrogen atmosphere, as shown in Figure 4b. Two degradation steps were clearly observed for the cross-linked binders. The first degradation step from approximately 180 to 350 °C was related to the decomposition of the imidazolium groups. The degradation step above 380 °C was attributed to the degradation of the main polymer chain. 

Figure 4c shows the residual weight when the temperature was increased to 1000 °C under TGA. The results confirmed that the remaining weight significantly increased with the increasing ratio of quaternized silane and thermal treatment up to 160 °C. The TGA analysis of the NiO@C-PVIm_Si is displayed in Figure S4.

**Figure 5:**
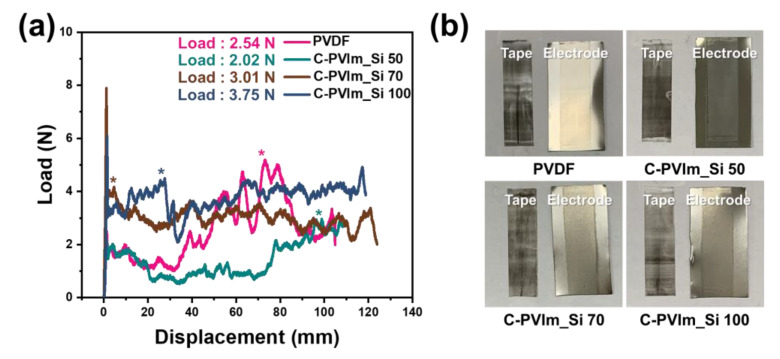
(**a**) The 180° peel tests for the Ni electrode with different binders; (**b**) optical images of the tape and electrode surface after the peel test.

The force–displacement curves of the Ni electrode, shown in Figure 5, indicate the adhesion between the binder and Ni electrode. The average peel forces of the C-PVIm_Si 50 (2.02 N), C-PVIm_Si 70 (3.01 N), and C-PVIm_Si 100 (3.75 N) binders were also higher than that of the PVDF (2.54 N) binder (Figure 5a).

Regarding the PVDF binder, uneven exfoliation was observed, and the strength was low. The PVIm_Si binder, on the other hand, showed uniform exfoliation. The results of the mechanical strength analysis confirmed that PVIm_Si produced a higher load value. This was assumed to result in improved adhesion to the electrode through the cross-linking of the PVIm_Si binder.

Moreover, the optical images showed that when the PVDF or C-PVIm_Si was used as a binder, a large portion of the electrode slurry peeled off from the Ni electrode and adhered to the tape side after the peel test (Figure 5b) [65].

Additionally, the solution concentration, the solution temperature in the reactor, and the complexing reagent of Ni^2+^ ions were important factors in preparing high-density spherical Ni(OH)_2_ with excellent performance.

**Figure 6:**
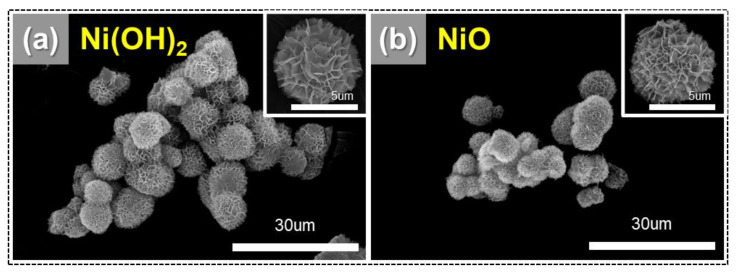
SEM images showing porous NiO nanostructure. (**a**) Ni(OH)_2_ and (**b**) NiO.

Thus, the concentration of the solutions, complexing reagent, feed-in velocity, intensity of agitation, temperature of the reactor, and pH value of the solution in the reactor were important for optimizing the performance of the product under controlled crystallization. The SEM image shows the external morphology of the Ni(OH)_2_ obtained through the synthesis, where the particles are spherical in shape (Figure 6) [66]. Elemental mapping by energy-dispersive X-ray spectrometry (SEM/EDS) was performed for Ni(OH)_2_ and NiO samples. Obtained compositions from the EDS analysis of individual samples were compared to the corresponding targeted chemical phase. The obtained compositions matched well within the typical errors related to EDS analysis. A representative EDS spectrum and elemental mapping is shown in Figure S5 a–j. The obtained data suggest the homogeneous dispersion of Ni, O, in both Ni(OH)_2_ and NiO (Figure S5 a–e, and f–j), respectively.

Porous NiO nanostructure powders were obtained by annealing the as-prepared Ni(OH)_2_ samples. After calcination, a color change from light green to gray was observed, confirming the conversion of Ni(OH)_2_ to NiO, as shown in Figure 6 [67]. Moreover, the XRD patterns of Ni(OH)_2_ and NiO are shown in Figure S6. The XRD peaks in sky blue can be identified as those of a Ni(OH)_2_, on the basis of the standard pattern in JCPDS card 22-044.

**Figure 7:**
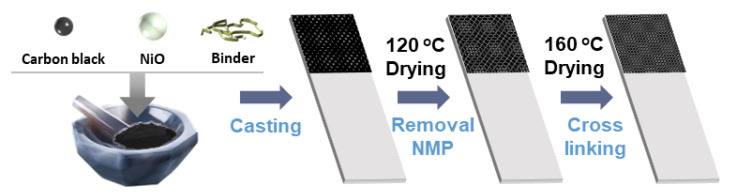
Schematic illustration of working electrode.

**Figure 8:**
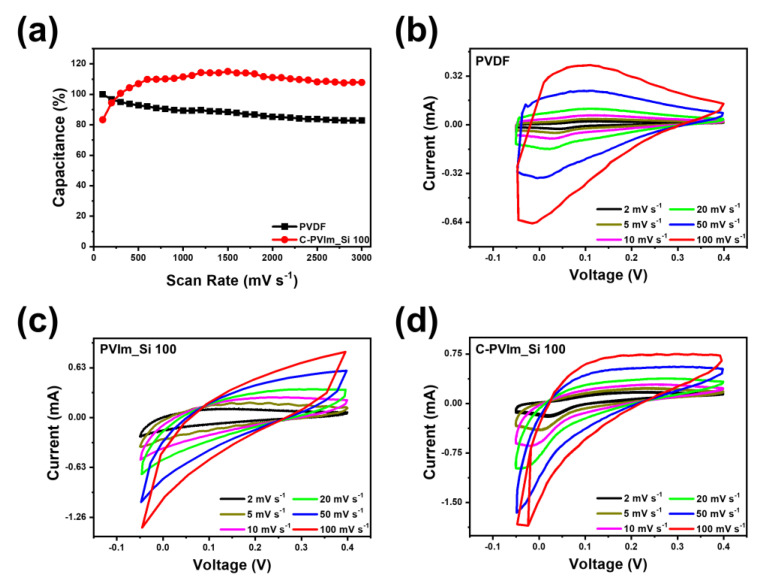
Cyclic voltammogram curve of capacitor at different scan rates of NiO. (**a**) Cycle test; (**b**) PVDF; (**c**) PVIm_Si 100; (**d**) C-PVIm_Si 100.

In this study, the working electrode was fabricated as shown in Figure 7. Based on this cross-linking study, the cyclic voltammogram (CV) test for NiO, the active material of supercapacitors, is as shown in Figure 8 [68,69,70]. To observe the change in the CV curve of the C-PVIm_Si 100 binder, which exhibited the best physical and thermal stability, different scan rates were obtained. In the Nyquist plot (Figure S7), the impedance behaviour of the Ni electrode is portrayed and shown by a vertical line in the low-frequency region, showing ideal capacitive behavior coupled with low charge transfer resistance (Rct). Similarly, the PVDF revealed a larger semicircle than C-PVIm_Si 100.

The smallest was observed for C-PVIm_Si 100 in the high-frequency region, which implied that C-PVIm_Si 100 had the lowest charge transfer resistance in comparison with others.

Our binder exhibited a higher capacity than the PVDF binder (Figure 8b). When comparing the capacities before and after cross-linking, a significant improvement in the capacity after cross-linking was observed (Figure 8c,d), confirming that the cross-linking rate affected the electrochemical capacity [71,72,73]. It was inferred that the durability was improved because the binding force between the electrode and binder was increased owing to the cross-linking reaction, revealing that the prepared binder showed a high capacity in PSEUDOs and had cycle stability. Therefore, we firmly believe that our proposed C-PVIm_Si binder has sufficient potential to replace PVDF.

## 4. Conclusions

A PVIm_Si binder was successfully synthesized to improve the cycle characteristics of PSEUDOs. This polymeric binder exhibited a strong binding ability with the electrode slurry and Ni electrode. ^1^H-NMR and FT-IR analyses confirmed the synthesis of PVIm_Si, and the thermal stability and cross-linking degree increased as the ratio of silane increased through TGA and gel content. Additionally, a peel test was performed to confirm the physical stability. Examining the electrochemical properties by manufacturing an electrode to which the C-PVIm_Si binder was applied revealed that the binding force between the electrode and binder was increased owing to the cross-linking effect. The resulting C-PVIm_Si 100 binder exhibited better adhesion and cycle stability than the PVDF binder. Thus, it was confirmed that the produced binder showed a high capacity in PSEUDOs and cycle stability. The C-PVIm_Si binder, which has advantages such as easy synthesis and excellent cross-linking, can be competitively used for its high adhesion and can also be applied to high-capacity electrode materials other than PSEUDOs. This study revealed that cross-linked networks in functional polymers are long-cycle-life features for electrochemical redox capacitors.

## Data Availability

Not applicable.

## Supplementary Materials

The following supporting information can be downloaded at: https://www.mdpi.com/article/10.3390/polym14235174/s1, Figure S1: FT-IR spectra of NiO@C-PVIm_Si.; Figure S2: ^1^H-NMR spectra. (a) PVIm; (b) 3CPTES; Figure S3: FT-IR spectra of C-PVIm_Si. (a) C-PVIm_Si 50 (150 ^o^C 1 h); (b) C-PVIm_Si 70 (150 ^o^C 1 h); (c) C-PVIm_Si 100 (150 ^o^C 1 h); (d) C-PVIm_Si 50 (150 ^o^C 2h); (e) C-PVIm_Si 70 (150 ^o^C 2h); (f) C-PVIm_Si 100 (150 ^o^C 2h); (g) C-PVIm_Si 50 (160 ^o^C 1 h); (h) C-PVIm_Si 70 (160 ^o^C 1 h) and (i) C-PVIm_Si 100 (160 ^o^C 1 h).; Figure S4: a) TGA Curves of NiO@C-PVIm_Si; b) final weight based on TGA.; Figure S5: SEM-EDS elemental map analysis of Ni(OH)_2_ and NiO. (a), (f) electron image; (b), (g) survey; (c), (h) O atoms; (d), (i) Ni atoms; (e), (j) EDS spectrum.; Figure S6: XRD patterns of Ni(OH)_2_ and NiO.; Figure S7: EIS spectra of the device showing the internal resistances PVDF and NiO@C-PVIm_100.
