# Supplementary material for: Ion-Conducting Robust Cross-Linked Organic/Inorganic Polymer Composite as Effective Binder for Electrode of Electrochemical Capacitor
Source: Polymers (Basel). 2022 Nov 28;14(23):5174. doi: 10.3390/polym14235174 (PMC9739130; doi:10.3390/polym14235174)
Supplement: Supplementary file 1 [file polymers-14-05174-s001.zip › polymers-2047361-SM.pdf]

polymers

MDPI

Article

Ion-Conducting Robust Cross-Linked Organic/Inorganic Polymer Composite as Effective Binder for Electrode of Electrochemical Capacitor

Hui Gyeong Park [1], Jin Ju Jeong [2], Jae Hun Kim [2], and Jung-Soo Lee [1,2,*]

[1] Department of Chemical Engineering, Graduate School of Chosun University, 309 Pilmun-daero, Dong-gu, Gwangju 61452, Republic of Korea

[2] Department of Bio-Chemical Engineering, Chosun University, 309 Pilmun-daero, Dong-gu, Gwangju 61452, Republic of Korea

* Correspondence: jslee15@chosun.ac.kr; Tel.: +82-62-230-7163; Fax.: +82-62-230-2474

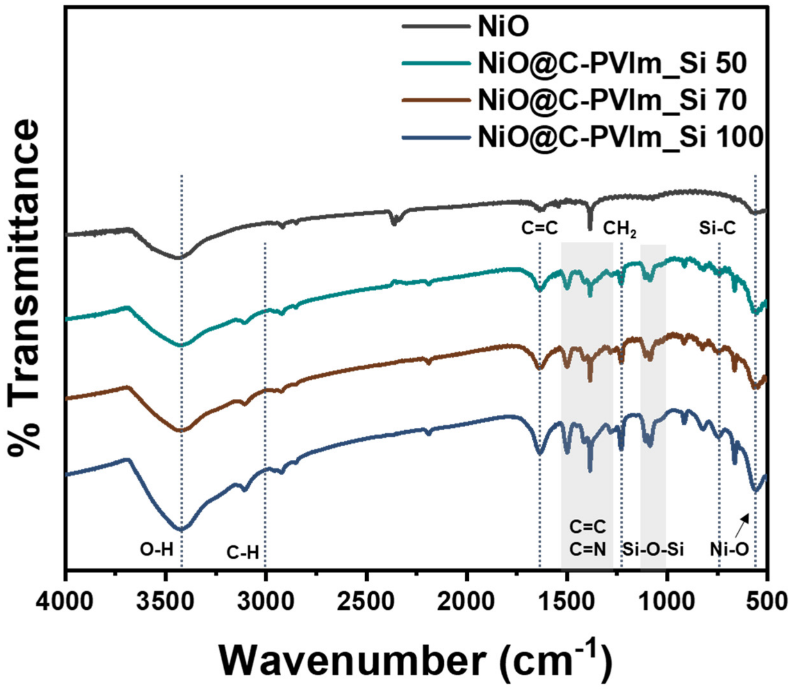

Figure S1. FT-IR spectra of NiO@C-PVIm_Si.

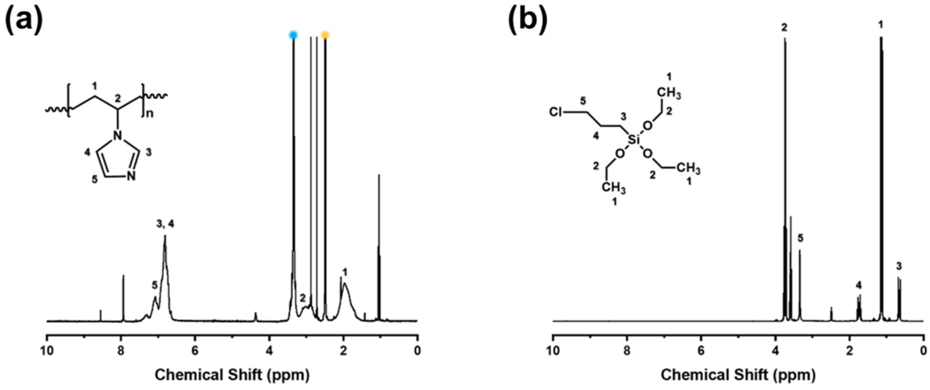

Figure S2. ^{1}H-NMR spectra of (a) PVIm; (b) 3CPTES.

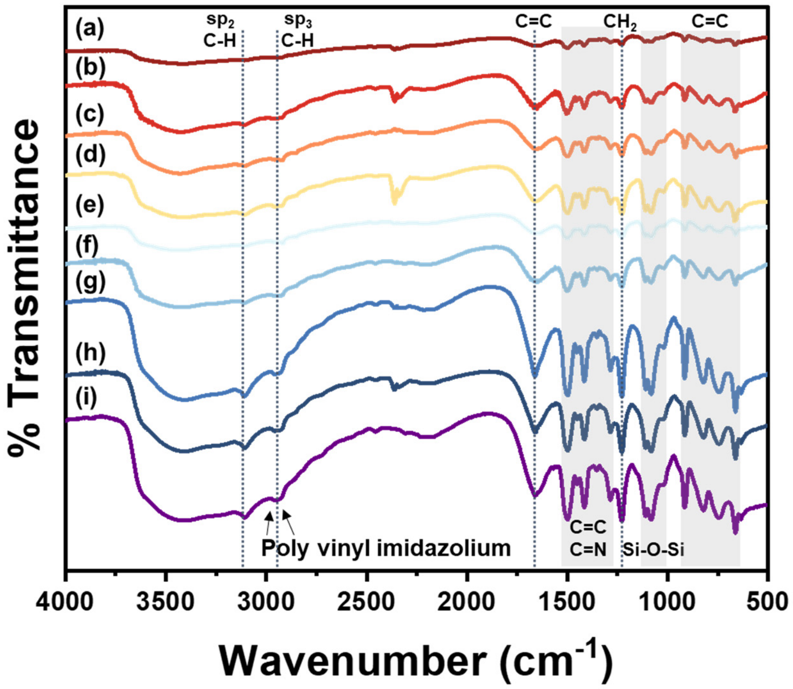

Figure S3. FT-IR spectra of C-PVIm_Si. (a) C-PVIm_Si 50 (150 °C 1h); (b) C-PVIm_Si 70 (150 °C 1h); (c) C-PVIm_Si 100 (150 °C 1h); (d) C-PVIm_Si 50 (150 °C 2h); (e) C-PVIm_Si 70 (150 °C 2h); (f) C-PVIm_Si 100 (150 °C 2h); (g) C-PVIm_Si 50 (160 °C 1h); (h) C-PVIm_Si 70 (160 °C 1h) and (i) C-PVIm_Si 100 (160 °C 1h).

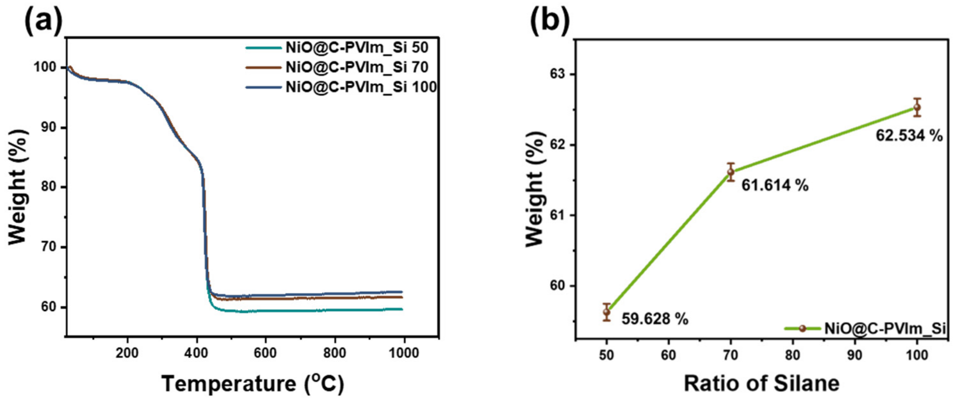

Figure S4. TGA Curves of (a) NiO@C-PVIm_Si; (b) final weight based on TGA.

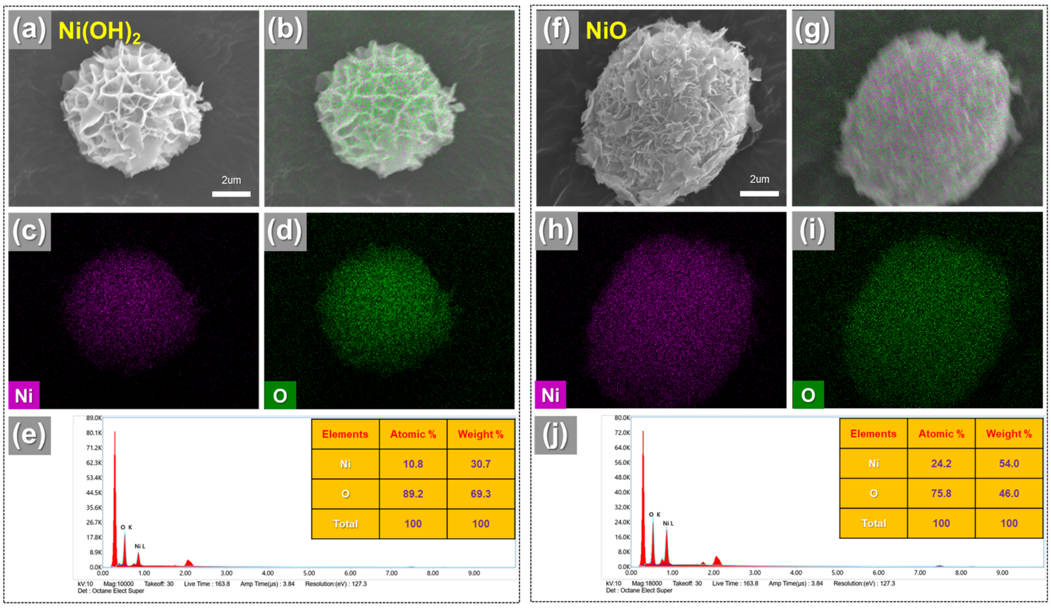

Figure S5. SEM-EDS elemental map analysis of Ni(OH)2 and NiO. (a), (f) electron image; (b), (g) survey; (c), (h) O atoms; (d), (i) Ni atoms; (e), (j) EDS spectrum.

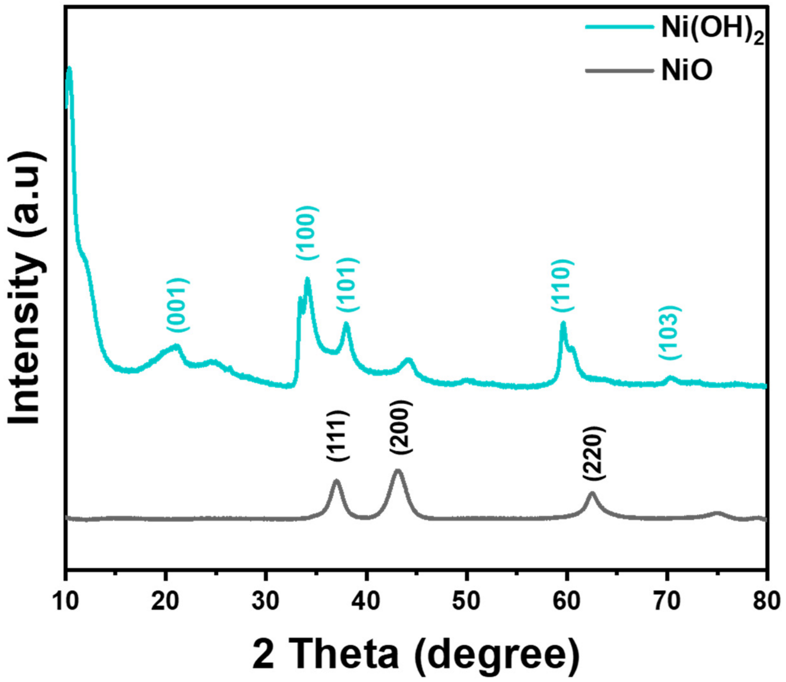

Figure S6. XRD patterns of Ni(OH)2 and NiO.

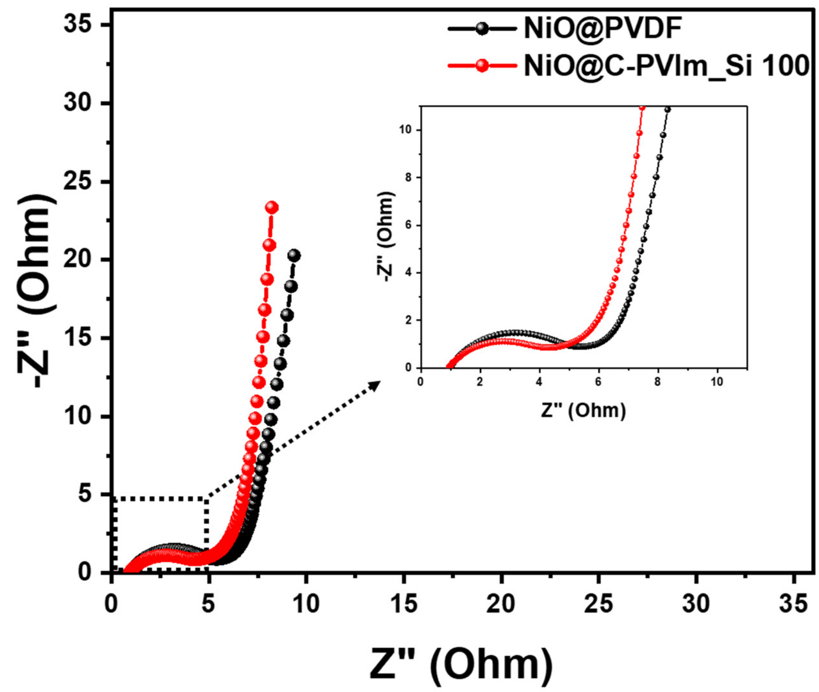

Figure S7. EIS spectra of the device showing the internal resistances PVDF and NiO@C-PVIm_Si 100.
